# Point-of-care ultrasound-guided fluid management in acute respiratory failure complicating preeclampsia and thyrotoxicosis: a case report

**DOI:** 10.11604/pamj.2026.53.82.51400

**Published:** 2026-02-13

**Authors:** Fransisca Dewi Kumala, Adhrie Sugiarto, Aino Nindya Auerkari

**Affiliations:** 1 Department of Anesthesiology and Intensive Care, Faculty of Medicine, Universitas Indonesia, Cipto Mangunkusumo National General Hospital, Jakarta 10430, Indonesia

**Keywords:** Point-of-care ultrasound, left ventricular outflow tract velocity time integral, preeclampsia, thyrotoxicosis, case report

## Abstract

The coexistence of preeclampsia and uncontrolled thyrotoxicosis leading to acute respiratory failure is rare and poses significant diagnostic and therapeutic challenges, particularly with respect to safe fluid management in the obstetric intensive care setting. We report the case of a 35-year-old woman at 37-week gestation who presented with progressive dyspnea, acute pulmonary edema, and fetal distress, necessitating emergency cesarean section and subsequent intensive care admission. Point-of-care ultrasound (POCUS) demonstrated impaired left ventricular systolic function (ejection fraction 40%), reduced left ventricular outflow tract velocity time integral (LVOT VTI 10-11 cm), a dilated inferior vena cava, and diffuse pulmonary B-lines, consistent with volume overload and compromised forward cardiac output. Serial POCUS-guided assessments were used to titrate diuretic therapy and optimize mechanical ventilation, resulting in progressive improvement in LVOT VTI (up to 20 cm), resolution of pulmonary edema, achievement of negative fluid balance, and clinical recovery. The patient was successfully extubated on day 5 and discharged home on day 7. This case demonstrates the clinical utility of POCUS, particularly serial LVOT VTI measurements, in providing actionable, dynamic bedside data to safely guide fluid management when static hemodynamic indices are unreliable, highlighting the value of POCUS in complex high-risk obstetric critical care.

## Introduction

Acute respiratory failure in the peripartum period represents a complex clinical challenge due to overlapping maternal and fetal considerations, where delayed diagnosis and inappropriate management may significantly increase morbidity and mortality [1]. Common etiologies include preeclampsia, pulmonary edema, peripartum cardiomyopathy, and endocrine disorders such as thyrotoxicosis [2]. Optimal management relies on early identification of the underlying cause, careful hemodynamic assessment, and coordinated multidisciplinary care. Point-of-care ultrasound (POCUS) enables real-time assessment of cardiac function, pulmonary congestion, and intravascular volume status, facilitating rapid and targeted clinical decision-making [3]. In obstetric patients, physiological cardiovascular adaptations limit the reliability of static preload indices such as central venous pressure. In contrast, serial measurement of left ventricular outflow tract velocity time integral (LVOT VTI) provides a dynamic and physiologically meaningful surrogate for stroke volume and cardiac output [4]. We report a critically ill obstetric patient with preeclampsia and uncontrolled thyrotoxicosis who developed acute respiratory failure, in whom serial POCUS-guided LVOT VTI monitoring was instrumental in guiding fluid management and optimizing clinical outcomes within a multidisciplinary care model.

## Patient and observation

**Patient information:** a 35-year-old Indonesian woman (gravida 6, para 3, abortus 2) at 37-week gestation presented to the emergency department with progressive dyspnea over two weeks, which acutely worsened to orthopnea in 24 hours before admission. She had a history of chronic hypertension managed with lifestyle modifications and a known diagnosis of hyperthyroidism. Antithyroid medication had been discontinued during pregnancy. There was no history of cardiac disease, diabetes mellitus, renal disease, or previous intensive care admission. The patient had received routine antenatal care; however, detailed documentation regarding adherence to antithyroid therapy during pregnancy was limited. There was no known family history of cardiovascular or endocrine disease. She did not smoke, consume alcohol, or use illicit substances. No relevant occupational or environmental exposures were identified. Before the current presentation, there had been no documented hospital admission during this pregnancy, and no prior interventions related to respiratory or cardiac symptoms were reported.

**Clinical findings:** on admission, the patient was in moderate to severe respiratory distress, with tachypnea (32 breaths/min), hypoxemia (SpO_2_ 89% on 15 L/min via non-rebreather mask), and hypertension (173/94 mmHg), and sinus tachycardia (heart rate 110 bpm). Fetal heart rate monitoring revealed fetal tachycardia (180 bpm). Physical examination of the chest demonstrated bilateral fine inspiratory crackles, consistent with pulmonary congestion.

**Diagnostic assessment:** laboratory investigations showed proteinuria, elevated free T4 (1.58 ng/dL), and suppressed TSH (<0.09 mIU/L). Chest radiograph revealed pulmonary edema with cardiomegaly.

**Diagnosis:** a diagnosis of acute respiratory failure with pulmonary edema secondary to preeclampsia and thyrotoxicosis, complicated by fetal distress, was made. Differentiating cardiogenic pulmonary edema from other peripartum causes of acute respiratory failure, including peripartum cardiomyopathy and fluid overload, was challenging due to overlapping clinical, radiographic, and hemodynamic features.

**Therapeutic interventions:** the patient underwent emergency cesarean section under general anesthesia and was admitted to the intensive care unit (ICU), intubated postoperatively. In the ICU, she was managed with lung-protective mechanical ventilation (FiO_2_ of 90%, positive end-expiratory pressure (PEEP) 9 cm H_2_O, tidal volume (Vt) of 6 mL/kg) to achieve SpO_2_ 95%. A point-of-care ultrasound was performed to further characterize cardiopulmonary status. Lung ultrasonography demonstrated diffuse bilateral B-lines, indicative of interstitial pulmonary edema. Focused cardiac ultrasound revealed impaired left ventricular systolic function with an ejection fraction (EF) of 40%, reduced left ventricular outflow tract (LVOT) velocity time integral (VTI) measuring 10-11 cm, a moderately dilated inferior vena cava (IVC) with reduced respiratory variation, and mild mitral regurgitation.

**Figure 1:**
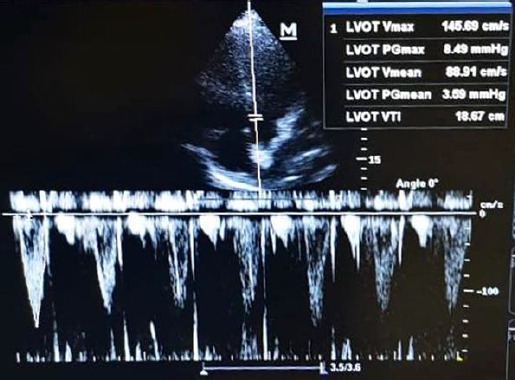
Doppler ultrasound showing left ventricular outflow tract velocity time integral waveform on intensive care unit day 2

**Figure 2:**
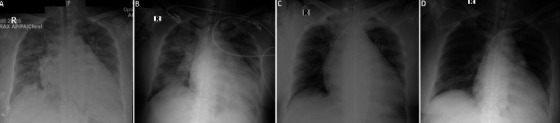
A) chest radiographs on arrival; B) intubated; C) 6 hours after intubation; D) intensive care unit day 4

These findings were consistent with volume overload and compromised forward cardiac output in the setting of acute respiratory failure. Central venous pressure (CVP) was 15 mmHg. Continuous IV furosemide (10 mg/h), thiamazole, and propranolol were initiated. Six hours later, repeat POCUS demonstrated improved LVOT VTI (13-14 cm). On day 2, FiO_2_ was reduced to 45% with PEEP 8 cmH_2_O. Point-of-care ultrasound showed reduced B-lines, increased LVOT VTI to 18 cm as shown in Figure 1, and less dilated IVC. Central venous pressure was 9 mmHg after a negative fluid balance of 500 mL in 11 hours; diuretic dose was tapered to 5 mg/h. On day 4, the cumulative fluid balance was negative 3200 mL. Point-of-care ultrasound showed an A-line profile, LVOT VTI of 20 cm, and a normalized IVC diameter. Chest radiograph confirmed resolution of pulmonary edema. Serial chest radiographs are shown in Figure 2. She was extubated after a successful spontaneous breathing trial and maintained oxygenation with a nasal cannula.

**Follow-up and outcome of interventions:** by the fifth day of care, the patient´s clinical status was stable, requiring only minimal oxygen support. She was discharged to the ward on day 6 and home on day 7 in good condition.

**Timeline of current episode:** the patient's clinical course, diagnostic evaluation, therapeutic interventions, and serial POCUS findings are summarized in Table 1. Serial assessments demonstrated a temporal association between negative fluid balance, incremental improvement in LVOT VTI, resolution of pulmonary edema, and recovery of respiratory function, ultimately leading to successful extubation and hospital discharge.

**Table 1:** timeline of clinical course, interventions, and point-of-care ultrasound findings

Day	Clinical status	Interventions	Point-of-care ultrasound findings	Fluid balance/monitoring
Admission (day 0)	Dyspnea, orthopnea, SpO_2_ 89%, BP 173/94, HR 110, FHR 180	Emergency cesarean section under GA	Not performed	-
ICU day 1	Intubated, ventilated FiO_2_ 90%, PEEP 9, SpO_2_ 95%	IV furosemide 10 mg/h, thiamazole, propranolol	B-lines, EF 40%, LVOT VTI 10-11 cm, dilated IVC, mild MR	CVP 15 mmHg; negative balance -500 mL (11 hours)
Six (6) hours post-admission	Oxygenation improving SpO_2_ 99%	Continued therapy	LVOT VTI ↑ to 13-14 cm	-
ICU day 2	Improving, FiO_2_ ↓ to 45%, PEEP 8, SpO_2_ 99%	Furosemide tapered to 5 mg/h	Reduced B-lines, EF 45%, LVOT VTI 18 cm, IVC less dilated	CVP 9 mmHg; negative balance -1500 mL
ICU day 3	Improving, FiO_2_ ↓ to 35%, PEEP 7, SpO_2_ 99%, wean to minimal ventilator support	Furosemide 3 mg/h	A-line profile, LVOT VTI 20 cm, IVC less dilated	CVP 7 mmHg; negative balance -1200 mL
ICU day 4	Stable, minimal ventilator support, awake → extubated	Furosemide 2 mg/h	A-line profile, LVOT VTI >20 cm, IVC normalized	CVP 6 mmHg; negative balance -700 mL
ICU day 5	Stable, minimal O_2_ support	Oral furosemide and ramipril were initiated	Further improved cardiac and lung profiles	CVP 4 mmHg; negative balance -500 mL
ICU day 6	Stable, no respiratory distress	Transition to the ward	-	-
Day 7	Stable, no respiratory distress	Discharged home	-	-

ICU: intensive care unit, GA: general anesthesia, LVOT VTI: left ventricular outflow tract velocity time integral, IVC: inferior vena cava, MR: mitral regurgitation, CVP: central venous pressure; ↓ indicates a decrease in the measured parameter; ↑ indicates an increase in the measured parameter; → indicates progression or transition to the next clinical stage

**Patient perspective:** patient reported marked relief of dyspnea, good functional recovery, and was satisfied with the treatment received following discharge from the ICU and hospital.

**Informed consent:** it was obtained from the patient for publication of this case.

## Discussion

This case illustrates the complex interaction between preeclampsia and uncontrolled thyrotoxicosis as contributory mechanisms for acute respiratory failure in pregnancy. Preeclampsia predisposes patients to pulmonary edema through endothelial injury, increased capillary permeability, and altered vascular tone [2,5], while thyrotoxicosis creates a hyperdynamic circulatory state that can further compromise cardiac function and exacerbate volume overload [6]. The coexistence of these conditions poses significant diagnostic and therapeutic challenges, particularly in distinguishing cardiogenic pulmonary edema from other peripartum causes of respiratory failure [6].

**Figure 3:**
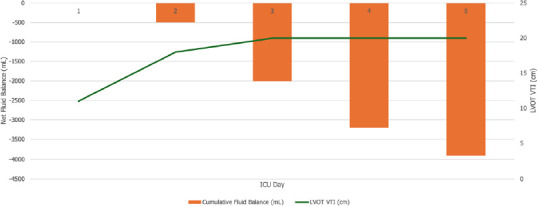
trend of cumulative fluid balance (mL) and left ventricular outflow tract velocity time integral (cm) during intensive care unit stay, demonstrating progressive improvement in cardiac output with guided diuretic therapy

Point-of-care ultrasound played a central role in the diagnostic evaluation and ongoing management of this patient. Initial POCUS findings demonstrated pulmonary congestion, impaired left ventricular systolic function, and reduced LVOT VTI, indicating compromised forward flow in the setting of volume overload. In contrast to static hemodynamic indices such as CVP, which are known to be unreliable in obstetric patients due to physiological cardiovascular adaptations, LVOT VTI provided a dynamic and physiologically meaningful surrogate for stroke volume and cardiac output [7]. Serial LVOT VTI measurements allowed real-time assessment of treatment response and informed safe titration of diuretic therapy. Figure 3 illustrates the trend of cumulative fluid balance and LVOT VTI throughout ICU stay. These findings underscore the utility of POCUS in tailoring treatment plans for critically ill patients.

The role of LVOT VTI as a surrogate for stroke volume and cardiac output has been validated in critical care environments. In contrast to static indices, LVOT VTI provides real-time, dynamic information on cardiac activity, allowing physicians to assess treatment responses and adjust fluid, vasoactive therapy, or ventilatory support accordingly [8,9]. In obstetric critical care, where fluid overload and hypovolemia may be harmful, POCUS offers a safe, reliable, and radiation-free modality to enhance maternal hemodynamics without increasing additional risk to the fetus.

The integration of cardiac and lung ultrasonography was particularly valuable in guiding therapeutic decisions in this case. The transition from diffuse B-line patterns to an A-line profile paralleled improvements in LVOT VTI, achievement of negative fluid balance, and clinical recovery. This multimodal ultrasound approach facilitated individualized fluid management while minimizing the risk of iatrogenic volume overload, a critical consideration in high-risk obstetric ICU patients. Despite its clinical utility, LVOT VTI remains underreported in obstetric critical care literature. Barriers include limited training in advanced echocardiographic techniques, concerns regarding feasibility in unstable peripartum patients, and continued reliance on traditional static measures. This case demonstrates that serial LVOT VTI assessment is feasible, informative, and clinically impactful when performed by trained operators within a multidisciplinary care framework.

Several limitations should be acknowledged. As a single case report, the findings may not be generalizable to all obstetric ICU populations. Additionally, LVOT VTI measurements are operator-dependent and require appropriate expertise to ensure accuracy and reproducibility. Nonetheless, the consistent temporal relationship between POCUS findings, therapeutic adjustments, and clinical improvement supports the validity of the management strategy employed.

## Conclusion

In critically ill obstetric patients with complex and overlapping pathologies, POCUS provides a valuable bedside tool for dynamic cardiopulmonary assessment. Serial LVOT VTI measurements can safely guide fluid and hemodynamic management by offering real-time insights into cardiac output and treatment response, particularly when static hemodynamic indices are unreliable. Incorporating multimodal ultrasound into multidisciplinary obstetric critical care may enhance decision-making and improve patient outcomes.
